# Crossing Kingdoms: How the Mycobiota and Fungal-Bacterial Interactions Impact Host Health and Disease

**DOI:** 10.1128/IAI.00648-20

**Published:** 2021-03-17

**Authors:** William Santus, Jason R. Devlin, Judith Behnsen

**Affiliations:** a Department of Microbiology and Immunology, University of Illinois at Chicago, Chicago, Illinois, USA; University of California, Santa Cruz

**Keywords:** mycobiome, mycobiota, fungi, interkingdom interactions, fungal-bacterial interactions, *Candida*, bacterial-fungal interactions, commensal fungi, host-pathogen interactions, microbiome, microbiota

## Abstract

The term “microbiota” invokes images of mucosal surfaces densely populated with bacteria. These surfaces and the luminal compartments they form indeed predominantly harbor bacteria. However, research from this past decade has started to complete the picture by focusing on important but largely neglected constituents of the microbiota: fungi, viruses, and archaea. The community of commensal fungi, also called the mycobiota, interacts with commensal bacteria and the host. It is thus not surprising that changes in the mycobiota have significant impact on host health and are associated with pathological conditions such as inflammatory bowel disease (IBD). In this review we will give an overview of why the mycobiota is an important research area and different mycobiota research tools. We will specifically focus on distinguishing transient and actively colonizing fungi of the oral and gut mycobiota and their roles in health and disease. In addition to correlative and observational studies, we will discuss mechanistic studies on specific cross-kingdom interactions of fungi, bacteria, and the host.

## Abstract (precis)

The term “microbiota” invokes images of mucosal surfaces densely populated with bacteria. These surfaces and the luminal compartments they form indeed predominantly harbor bacteria.

## INTRODUCTION

## WHAT IS THE MYCOBIOTA?

**FIG 1:**
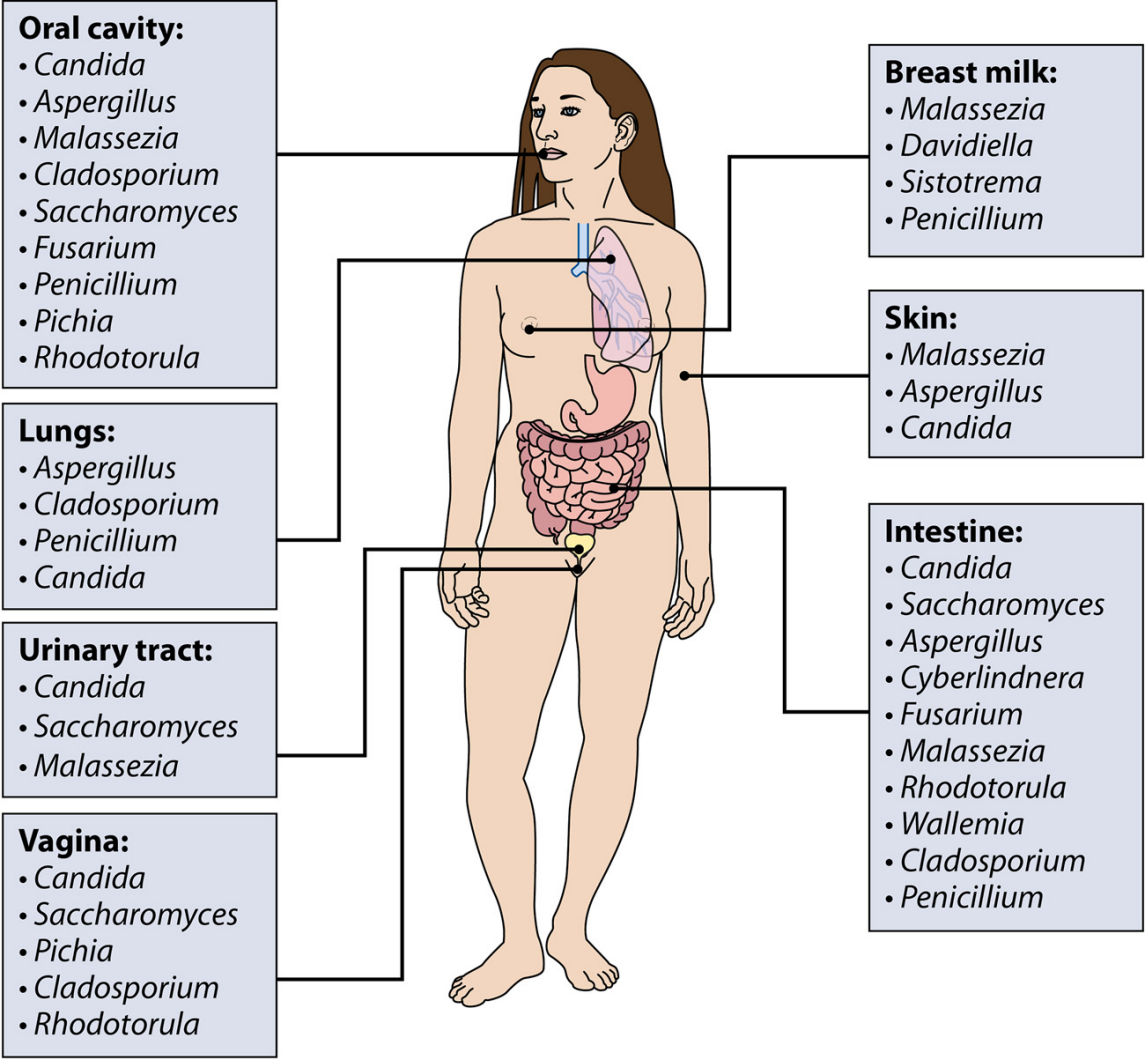
Fungal populations in and on different anatomical sites. Fungal populations have been identified in and on almost all human body sites. This figure is a schematic representation of the most commonly identified fungal genera under nonpathological conditions in the oral cavity (53, 54, 56, 57, 184), skin (92, 103), urinary tract (6, 7), vagina (4, 185), breast milk (93), lungs (186, 187), and intestine (44, 96, 104, 188–190).

Fungi are microeukaryotes that can be found on various mammalian mucosal surfaces, such as the lungs (1–3), the vaginal tract (4, 5), the urinary tract (6, 7), the oral cavity (8–10), and the intestines (9, 11, 12) as well as on the skin (5, 9, 13–15), breast, and in breastmilk (16) (Fig. 1). Historically, research on fungi focused on pathological conditions and fungi as pathogens or pathobionts. For example, expansion of the commensal yeast *Malassezia* on the skin is associated with a disease known as pityriasis versicolor (17). Inhaled spores of the mold Aspergillus fumigatus can germinate and cause invasive lung disease in immunocompromised patients (18, 19). The yeast Candida albicans is arguably the most-studied pathogenic fungus and is responsible for a variety of disease conditions, which include vulvovaginal candidiasis in women, oropharyngeal candidiasis in infants and immunocompromised patients, and invasive candidiasis with systemic dissemination of *Candida* to peripheral organs (20–23).

Fungi not only are the causative agents of disease but also can be isolated from mammals in the absence of disease (11, 15, 24–26). C. albicans, for example, can frequently be isolated as a commensal of the oral cavity, vagina, or gut of healthy individuals and only causes infections if the host immune system is compromised or the local microbiota is disturbed (22, 23). However, a culture-dependent approach has a high likelihood to yield an incomplete picture of the total fungal diversity (11, 12). The advent of sequencing technology allowed us to answer important questions about fungi: do complex commensal communities of fungi exist in or on different mammalian anatomical sites? Which fungi do they comprise? Are they transiently present or do they stably colonize? What are their functions? The last decade has seen a stark increase in publications addressing these questions. We now know that diverse commensal fungal communities exist in and on mammals. These fungal communities are commonly referred to as the fungal microbiota or mycobiota and are the subject of exciting new research.

## METHODS TO ANALYZE THE MYCOBIOTA

A variety of different methods have been used to detect live fungal cells or fungal genomes. Some of the techniques include direct culturing, enriched culturing, microscopy with fluorescence *in situ* hybridization (FISH) or immunofluorescence, flow cytometry, amplicon sequencing (e.g., internal transcribed spacer [ITS]), and whole-genome shotgun sequencing. However, there are distinct advantages and disadvantages to each approach. Therefore, a combination of different methods described in the following paragraphs will help solve important current questions regarding what constitutes a core mycobiota and which fungi are transient or resident.

### Culturing of fungi.

Many environmental, commensal, and pathogenic fungi can be cultured on standard media (11, 25). However, some fungi found in mammals require specific medium conditions. *Malassezia* species, for example, fail to grow in the absence of fatty acids (17), and anaerobic fungi from ruminants require an anaerobic environment and specific additions such as wheat straw for culture (27). A broad “culturomics” approach identified the highest number of fungi from human gut samples on Dixon medium, a complex medium that includes malt extract, ox bile, and different fatty acids (11). In the gut, bacteria greatly outnumber fungi, which comprise approximately 0.1% of the gut microbiota (28). Isolation of fungi from gut samples therefore usually requires the addition of antibiotics (11, 25). Nevertheless, fungal species with a low abundance might not be recovered. Colonies can be identified to the species level by species-specific PCR or amplification and sequencing of the ITS regions (11, 25). Species can also be identified via matrix-assisted laser desorption ionization–time of flight (MALDI-TOF) analysis, but identification is limited by the available databases, which are focused on pathogenic rather than commensal species (29, 30). One of the drawbacks of microbial culture is that it will identify all viable fungi, including fungal spores or transiently present fungi that might not be metabolically active in the gastrointestinal tract.

### Visualization of fungi.

Culturing identifies viable organisms in a given sample. However, unless sampling is performed in specific sections (e.g., mucosa versus lumen), it gives no spatial information. Staining for fungi in fixed or frozen tissue samples can provide such spatial information. Fungi can be visualized in sectioned samples via immunostaining with fungal specific antibodies (12, 31), soluble conjugated receptors (12), or fluorescence *in situ* hybridization (32, 33). These approaches are limited by the specificity of antibody or probe used. However, they will identify fungal cells in a given sample and omit relic DNA.

### Metagenomic analyses.

The specific technical and bioinformatic demands of mycobiome sequencing are expertly outlined elsewhere (34–36). Sequencing detects DNA of fungi present in a given sample regardless of whether they are culturable or not. Amplicon sequencing uses fungal specific primers to amplify ITS or 18S regions of the rRNA gene locus, which contain hypervariable domains and allow for species discrimination (12, 37–39), analogous to 16S bacterial sequencing. This method illuminated a diverse mycobiome in humans and virtually all other species analyzed, for example, mice (12, 32, 40–44), pigs (45), dogs (46), bees (47), and lizards (48). We will discuss the most frequently identified fungi of the human and mouse oral and gut mycobiomes in the following sections. Shortcomings of amplicon sequencing include amplification bias and the lack of comprehensive and fully annotated reference databases that take the complex fungal taxonomy into account (35, 39). Whole-genome shotgun sequencing does not require amplification and can be used to analyze bacterial and fungal metagenomes simultaneously (28). An advantage is that publicly available data sets generated for bacteriome analysis can be reanalyzed for fungi (49). However, the presence of fungi might be underestimated, since fungal sequences are vastly outnumbered by bacterial sequences and some are not accurately identified as fungal due to the scarcity of available fungal genomes (36). A general drawback of DNA sequencing-based methods is the inability to discern between metabolically active organisms and relic DNA (36). The amount of relic DNA content in human feces seems currently unknown, but relic DNA accounts for >40% of recovered sequences from soil samples (50). In the future, amplicon sequencing of the fungal ITS region derived from total RNA (36) or metatranscriptomic approaches might help to address this important question (51).

## THE ORAL MYCOBIOTA

The gateway to the gastrointestinal tract is the oral cavity. Interactions of oral mycobiome members influence the local environment but can also have more distant effects. For example, C. albicans abundance in fecal matter is diminished when oral C. albicans abundance is reduced by more frequent brushing of teeth (52). Here, we will address which members of the oral mycobiota might constitute a core mycobiome, identify which are transient and which are colonizing, and highlight examples of interactions with fungi occurring in the oral cavity.

### Members of the oral mycobiota.

In their seminal study, Ghannoum and colleagues identified a total of 101 species in the oral cavities of 20 healthy individuals. However, 39 genera were present in only one subject and just 15 genera, including *Candida* and *Cladosporium*, were present in more than four individuals (53) (Fig. 1). Another study aimed to define the oral mycobiome found *Malassezia* to be the most prevalent genus in the oral mycobiomes of six different healthy individuals (54). The oral mycobiome thus appears to be more subject specific than the oral bacteriome, where 47% of bacterial operational taxonomic units (OTUs) were shared between three analyzed samples (55). Despite the high interindividual variability, the core oral mycobiome within individuals seems largely stable, as it maintained a similar composition and relative abundance in human subjects over the course of a 30-week-long study (56). A recently published review article summarized the fungal species identified in different studies with both culturing and sequencing techniques (57). Interestingly, many of these fungi are known to be associated with plants, such as *Aureobasidium*, *Fusarium*, and *Alternaria*, and food commonly consumed, i.e., *Saccharomyces* and *Penicillium*, or found as mold both indoors and outdoors, such as *Aspergillus* species and *Cladosporium* (Fig. 1). This poses the question if all identified fungi are actively colonizing or if some are transiently present.

### Active and transient colonizers.

**FIG 2:**
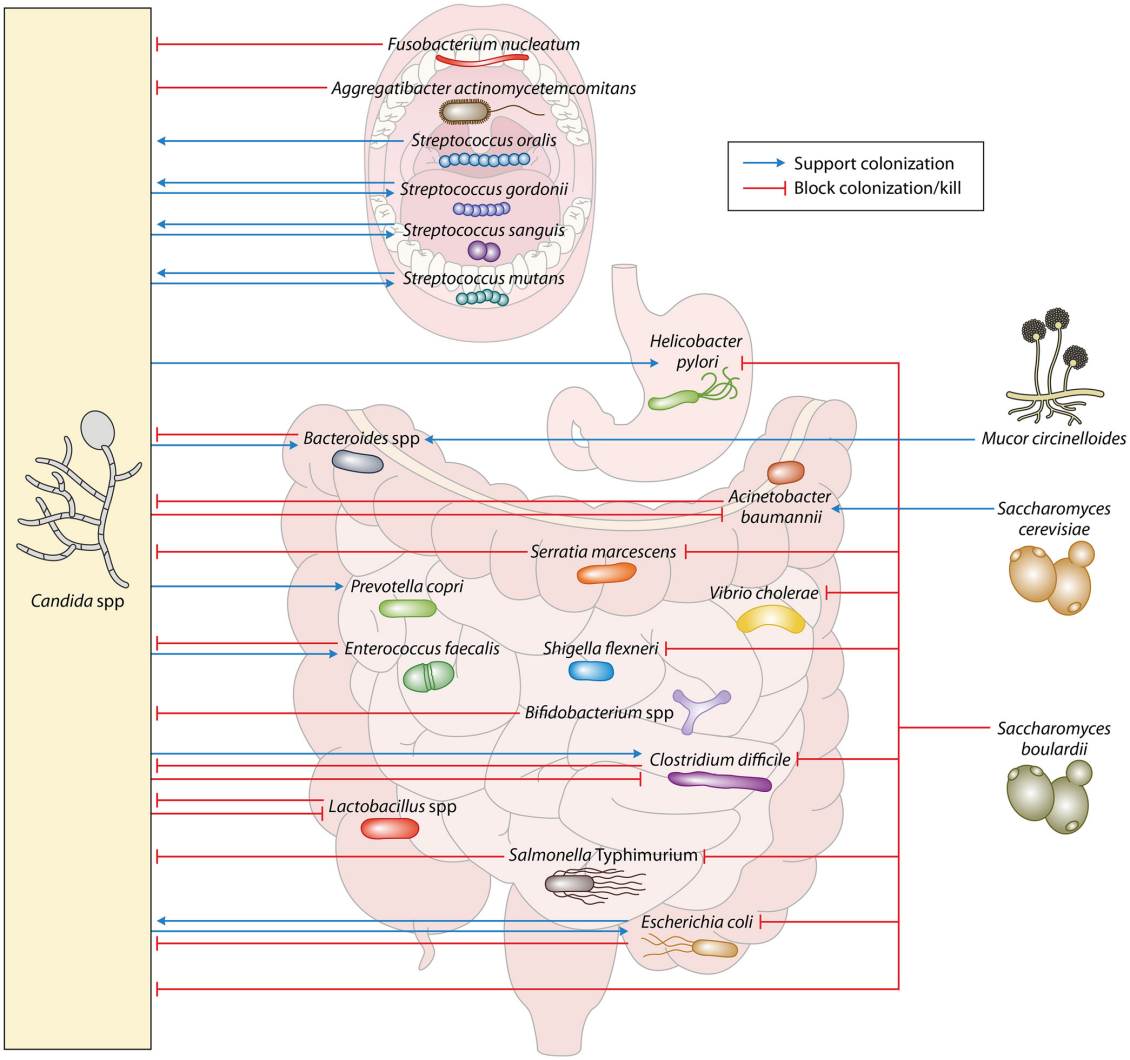
Specific fungal-bacterial interactions in the gastrointestinal tract. Schematic representation of the bacteria-fungi interactions discussed in this review. Localization in the cartoon is not a representation of where the interactions occur within a specific organ. Fungi are depicted outside the organs for schematic purposes.

Among the most commonly found fungi in the oral cavity are *Aspergillus* species, e.g., A. niger, and *Candida* species, such as C. albicans, C. tropicalis, and C. parapsilosis (58). A recent study identified them as the most abundant species in both healthy individuals and patients with periodontal disease (59). *Aspergillus* species are spore-forming filamentous fungi ubiquitously present in the environment (60). Even though *Aspergillus* species have been found as members of the oral mycobiome, pathological conditions due to *Aspergillus* are rare in the oral cavity and are usually restricted to the lungs and respiratory tract (61). On the other hand, *Candida* infections of the oral cavity are very common and affect up to 7% of infants, 30% of HIV patients, and 20% of cancer patients (58). This discrepancy might be due to the possibility that *Aspergillus* is a transient member of the oral mycobiome, acquired via diet intake or inhalation. A report from 1966 showed that Aspergillus flavus was cultured from 60% of analyzed wheat flours and comprised 5.8% of the total fungal load (62). *Candida* species actively colonize the oral cavity and erupt in infections when conditions allow it. Both culture-dependent and culture-independent studies have identified *Candida* species as components of the oral mycobiome. C. albicans was present in 90% (63–65) or even 100% of analyzed subjects (66). Older age, poor oral hygiene, and a fewer number of teeth are some of the associations found with an increased level of colonization of *Candida* species (53). C. albicans has also been shown to take an active part in biofilm formation and plaque virulence in combination with Streptococcus mutans (67) (Fig. 2). The oral mycobiome thus consists of both resident fungi and fungi that might only be transiently present. Mechanistic research on fungi in the oral cavity has been focused on the resident yeast C. albicans, which will be outlined in the next section. Other fungi of the oral mycobiota, such as *Saccharomyces*, *Aspergillus*, *Penicillium*, and *Malassezia*, might also represent active members of the oral microbiota, but their function has yet to be identified (53, 56).

### Examples of fungal-bacterial interactions in the oral cavity.

**(i) Protective interactions.** Most of the studies investigating the cross-kingdom interactions of fungi and bacteria in the oral cavity have focused on models involving C. albicans. Usually existing in its commensal yeast form, C. albicans can be stimulated to form invasive hyphae (68). The oral commensal Fusobacterium nucleatum has been shown to adhere to both the yeast and hyphal forms of C. albicans (69, 70). This interaction limits C. albicans hyphal formation, thus reducing its ability to kill macrophages *in vitro* (71). The commensal Aggregatibacter actinomycetemcomitans inhibits biofilm production by C. albicans through the secretion of the quorum-sensing molecule autoinducer-2 (72) and limits polymicrobial biofilm formation by C. albicans and Streptococcus mutans *in vitro* (73) (Fig. 2). The oral bacterial microbiota thus has an active role in limiting the conversion of C. albicans yeast cells to invasive hyphae.

**(ii) Pathogenic interactions.** Analysis of the salivary microbiomes of older adults revealed that *Candida* species abundance is associated with decreased bacterial diversity and increased abundance of *Streptococcus* species (74). Various studies have investigated mutualistic interactions between C. albicans and species of *Streptococcus* that promote infection. Glucose-starved C. albicans has been shown to coaggregate with multiple *Streptococcus* species, including S. sanguis, S. gordonii, and S. oralis (75) (Fig. 2). Further studies revealed that this coaggregation is mediated by cell wall polysaccharides, salivary proteins, and adhesins on the surface of S. gordonii (76–78). Adhesion is also mediated by the receptors Als1p, Als3p, and Als5p on the surface of C. albicans (79, 80). An *in vitro* study demonstrated that S. gordonii can enhance C. albicans biofilm formation (81, 82) (Fig. 2). S. mutans also enhanced biofilm production in the oral cavity of infected mice through interactions between a glucosyltransferase secreted by S. mutans and surface mannan expressed by C. albicans (67, 83). Another study used an oral infection model of immunosuppressed mice to show that coinfection with S. oralis enhances mucosal invasion of C. albicans by synergistically promoting E-cadherin degradation (84). These studies demonstrate that some members of the oral bacterial microbiota, specifically the genus *Streptococcus*, facilitate fungal overgrowth. More interactions between C. albicans and *Streptococcus* species have been reviewed in detail in reference 85.

## THE GUT MYCOBIOTA

The vast majority of microorganisms in mammals can be found in the gut, and fungi have emerged as an important component of the gut environment. Much research has therefore focused on understanding how the gut mycobiota is shaped and its interaction with human health.

### Development of the gut mycobiota.

The formation of the human mycobiota begins very early in life. Willis and colleagues recently suggested that fungal species might be present prior to birth and that C. albicans specifically could be associated with preterm delivery (86). Additional studies have shown that vaginal delivery allows vertical transmission of *Candida* species from mother to infant (87, 88). Infants born by C-section harbor a bacterial microbiome similar to the mother’s skin microbiome (89). They could therefore also harbor higher *Malassezia* species in their gastrointestinal tract, as this genus is the major colonizer of the human skin (90–92) (Fig. 1). After delivery, the gastrointestinal mycobiota is modulated by diet intake. For many infants, the main food source during the first months of age is breast milk. Boix-Amorós and colleagues found a core breast milk mycobiome, composed of *Malassezia*, *Davidiella*, *Sistotrema*, and *Penicillium*, that was shared by study participants despite a varied geographical origin (93). Accordingly, the infant gut mycobiome is initially dominated by *Malasseziales*, most likely taken up through lactation. After the first 6 months of age, the infant gut mycobiome undergoes a dramatic change and is no longer dominated by *Malasseziales* but by *Saccharomycetales* instead (94). This change in mycobiome coincides with a change from breast milk to solid food. The gut microbiota further changes and matures during the development from childhood to adulthood (95). These changes are most likely driven by the development of the immune system and by the microorganisms that humans are exposed to through their diet and environment. Similarly to oral mycobiome research, recent gut mycobiome research has focused on determining which fungal species are transient colonizers and which species are residents of the gastrointestinal tract.

### Active and transient colonizers.

Analysis of the Human Microbiome Project data determined *Saccharomyces*, *Malassezia*, *Candida*, and *Cyberlindnera* as the four most abundant genera present in the human gut (Fig. 1). However, researchers found very high mycobiome variability between individuals and within individuals over time (96–98). Compared to the bacterial gut microbiome, the gut mycobiome thus seems to be less consistent and stable over time (96). The high fluctuation might in part be explained by fungi being introduced in the gastrointestinal lumen via diet intake and environment. Indeed, a standard human diet contains high levels of live fungi and fungal DNA (62, 99–102), and some fungal genera identified in the human gastrointestinal (GI) tract are thought to lack the ability to grow at the temperature, pH, and low oxygen present in the gut environment (100). *Saccharomyces* is ubiquitously present in the human diet, while *Malassezia* is the most abundant fungus colonizing the human skin (92, 103). *Cyberlindnera* is a food additive and most likely acquired through the diet (96), while *Candida* is the most identified fungus in the oral cavity (104). Indeed, a small-scale study showed that presence of *Saccharomyces* in human feces was directly dependent on diet intake, while, as previously mentioned, C. albicans was associated with oral hygiene (52).

Current data thus suggest that some fungi in the human gastrointestinal tract can be classified as transient. However, evidence for true colonizers can also be found. C. albicans, Malassezia restricta, Cryptococcus neoformans, and others have been shown to bloom in the gut under inflammatory conditions. This is particularly highlighted in patients affected with inflammatory bowel disease (IBD), where C. albicans increases in abundance during inflammation. However, it is not yet clear if C. albicans creates the inflammatory environment or if its increase in abundance is a consequence of inflammation (12, 105, 106). A unique phylum of resident fungi can be found in some herbivores but is not detected humans or mice. *Neocallimastigomycota* are strictly anaerobic fungi that are present in all foregut fermenters (e.g., cows) and some hindgut fermenters (e.g., elephants), where they aid in the digestion of lignocellulose (107). Despite being an anaerobic environment, the human gut does not seem to support the colonization of strictly anaerobic fungi.

So, what proportion of the mycobiota is transiently present and what represents true residents? The bulk of research beginning to address this question has been performed in the mouse model, which we will focus on in the following section. Fungi in mice have been predominantly identified with culture-independent techniques (12, 32, 33, 40–44, 104). Interestingly, fungal sequences can be detected not only in feces but also in mouse chow (12, 32, 41). Some of the identified genera, such as *Aspergillus*, *Cladosporium*, and *Alternaria*, can be found in both feces and chow. However, other fungi, e.g., *Candida*, *Fusarium*, and *Saccharomyces*, can be found only in feces and not in chow (12, 32) or their abundance is expanded in feces compared to that in chow (41). Similarly, a more recent study found that 80% of the fungal taxa identified in mice fed a standard diet were not present in the diet and that 90% of fungal taxa identified in mice fed a high-fat diet were absent in the respective diet (40). These studies thus suggest that the composition of the mycobiota in the gut distinctly differs from the fungi present in the diet.

Even though culture-independent techniques identified fungal species unique to feces, reports showing fungi cultured from mouse feces are rare and restricted to non-specific-pathogen-free (non-SPF) conditions (43, 108). Possible reasons include (i) the relatively low abundance of fungi compared to that of bacteria or (ii) the sampling site, as feces might harbor a different composition of live fungi compared to that in sites in the upper gastrointestinal tract (109). Interestingly, laboratory mice released in an outdoor environment show an increased alpha-diversity of the mycobiome, and researchers were able to culture several fungal species from the mice’s feces. Most of the fungi that researchers were able to culture were *Aspergillus* species (43). This increase of fungi found in rewilded mice could be due to the presence of spores passing through the GI tract and/or an indication of live *Aspergilli* that have colonized the gut. The SPF environment of most laboratory mice might therefore be “too clean” to allow for the acquisition of living fungi to colonize their gut. This is supported by the discovery that 22% of laboratory mice do not survive cohousing with pet store mice (110). Pet store mice harbor bacteria that are absent in laboratory mice, and this might also be true for the fungal component of the microbiota. Collectively, studies in mice and humans support the idea that two mycobiomes are present in the mammalian gut: a transient mycobiome originating from diet intake and a resident mycobiome with persistently colonizing fungi. However, discriminating between transient fungi and active colonizers is still challenging. A combination of the different techniques outlined in Methods to Analyze the Mycobiota, as well as other parameters, i.e., activation of the host immune response and interaction between bacteria and fungi, as postulated by Fiers and colleagues (102), will be essential to characterize the role of transient and resident fungi in the gut.

### Bacterial-fungal interactions in the gastrointestinal tract.

Recent studies have suggested that the mycobiota plays roles in maintaining homeostasis of the bacterial microbiota and influencing overall gut health. One study found that the administration of antifungal drugs to dextran sulfate sodium (DSS)-treated mice exacerbates colitis and induces changes in the microbiome. Here, the microbiome undergoes an expansion of the bacterial genera *Hallella*, *Barnesiella*, *Bacteroides*, *Alistipes*, and *Lactobacillus* and a reduction of *Clostridium XIVa* and *Anaerostipes* (41). Another group found that the ingestion of the pathogenic fungus Mucor circinelloides by mice induced changes in the microbiota, notably with an increase in the genus *Bacteroides* and a decrease in *Akkermansia* (111). Another study demonstrated that C. albicans affects the recolonization of the cecum by the microbiota in mice treated with antibiotics. The presence of the fungus increased the recovery of bacterial diversity, specifically the return of *Bacteroides* species. However, it also allowed colonization by the pathobiont Enterococcus faecalis and reduced colonization of probiotic *Lactobacillus* strains (112) (Fig. 2). The mechanism of how C. albicans influences bacterial colonization is still unclear. A follow-up study revealed that antibiotic-treated C. albicans-colonized mice showed reduced expression of specific immune genes but no visible changes in inflammation. These changes in expression could be limiting the host’s ability to maintain microbial homeostasis, but there is still a possibility that C. albicans directly interacts with bacteria (113). A study that investigated differences in the microbiome between Japanese and Indian individuals proposed an interesting diet-fungal-bacterial interaction. The microbiome of the Indian participants showed a higher abundance of *Candida* and *Prevotella.* Since plants make up a major part of Indian diets, Pareek and colleagues (114) went on to show that arabinoxylan, a plant polysaccharide, can be used as a growth factor by various *Candida* species. Finally, they showed that *Candida* supernatant enhances the growth of Prevotella copri and that prior colonization by C. albicans is required for the colonization of germ-free mice by *P. copri* (114). These studies indicate that interactions between fungi and bacterial species influence gut homeostasis and are relevant to human health.

### Protective interactions.

Specific cross-kingdom interactions between fungi and bacteria are currently being explored as a tool to maintain intestinal homeostasis. The yeast Saccharomyces boulardii has been extensively studied as a potential probiotic due to its protective effect against various bacterial gastrointestinal pathogens, including Clostridium difficile, Helicobacter pylori, Vibrio cholerae, Salmonella enterica serovar Typhimurium, Shigella flexneri, and Escherichia coli (115–122) (Fig. 2). Protection against C. difficile is at least partially due to the production of a protease by *S. boulardii* that degrades toxins A and B of C. difficile (123, 124). Protection against V. cholerae seems to involve the recognition of cholera toxin and subsequent activation of cyclic AMP signaling by *S. boulardii* (125). Even though *S. boulardii* has shown efficacy in a rat model of V. cholerae infection (119), this has yet to show clinical significance for humans (126). Both E. coli and *S.* Typhimurium bind to the surface of *S. boulardii*, potentially preventing adhesion to intestinal epithelial cells and thus allowing quicker excretion through fecal matter (127, 128). This interaction is inhibited by the addition of exogenous mannose, indicating that E. coli and *S.* Typhimurium are adhering to surface mannose residues present on *S. boulardii* (129). *S. boulardii* may also interact with commensal *Enterobacteriaceae* to alleviate DSS-induced colitis, as this protective effect is lost in mice treated with *Enterobacteriaceae*-depleting antibiotics (130). The depicted interactions underline the antipathogenic potential for commensal fungi, but the inverse also occurs, where commensal bacteria can protect against pathogenic fungi.

The most intensely studied examples of pathogenic fungi being antagonized by commensal bacteria involve C. albicans. Four probiotic strains, Lactobacillus acidophilus, Lactobacillus reuteri, Lactobacillus casei GG, and Bifidobacterium animalis, have shown efficacy in limiting the severity of C. albicans infection in both immunocompromised and germ-free mice (131) (Fig. 2). Another probiotic mixture, consisting of *S. boulardii*, L. acidophilus, Lactobacillus rhamnosus, and Bifidobacterium breve, successfully inhibited the *in vitro* formation of polymicrobial biofilms containing E. coli, Serratia marcescens, and either C. albicans or C. tropicalis (132). These polymicrobial biofilms may be relevant to intestinal disease, as E. coli, S. marcescens, and C. albicans have shown higher abundance in fecal samples from Crohn’s disease patients (133). Various bacterial species have been shown to inhibit the transition of C. albicans to its invasive hyphal form. The widely studied probiotic L. rhamnosus GG produces an exopolysaccharide that limits hyphal formation and blocks C. albicans binding to intestinal epithelial cells *in vitro* (134). L. rhamnosus GG also inhibits C. albicans hyphal formation in liquid medium via the peptidoglycan hydrolase MspI, which degrades chitin present in the cell wall (135). Enterococcus faecalis produces the bacteriocin EntV to inhibit C. albicans hyphal formation, reducing pathogenicity in a murine oropharyngeal candidiasis infection model (136). Studies have also reported that soluble factors produced by E. coli show antifungal activity against C. albicans. A soluble factor from the E. coli K-12 strain induced the death of C. albicans *in vitro* (137), and supernatant from an E. coli biofilm inhibited biofilm formation on polystyrene plates for a variety of *Candida* species (138). Furthermore, metabolites produced by a consortium of bacterial species derived from healthy human fecal samples effectively inhibited the growth of C. albicans in liquid culture. Species of *Roseburia* and Bacteroides ovatus were directly responsible for these antifungal effects (139). Interestingly, C. albicans also demonstrates probiotic properties by enhancing the growth of two strictly anaerobic commensal bacteria, Bacteroides fragilis and Bacteroides vulgatus, in liquid media. Possible mechanisms of this interaction are utilization of surface mannan as a carbon source or reduction of culture oxygen levels by C. albicans (140). Beneficial interactions between bacteria and fungi are continuously being explored as potential probiotic interventions for intestinal disease.

### Pathogenic interactions.

Alternatively, interactions between fungal and bacterial commensals and pathogens have the potential to enhance pathogenesis. For example, mice treated with DSS to induce colitis showed increased disease when C. albicans was present. However, when mice were administered colistin to eliminate resident *Enterobacteriaceae*, the presence of C. albicans did not exacerbate colitis severity. Supplementation with colistin-resistant E. coli restored the C. albicans effect on DSS-induced colitis, suggesting that *Enterobacteriaceae* are required for C. albicans-mediated enhancement of colitis (130). Other studies have found that enterohemorrhagic E. coli enhances C. albicans invasion of intestinal epithelial cells *in vitro* (141). There is also evidence that E. coli strain O111:B4 enhances C. albicans infection of mice (142). E. coli 07KL was also found to enhance C. albicans attachment to epithelial cells *in vitro*, with a mechanism that likely involves bacterial pili (143). As mentioned in the previous section, the guts of Crohn’s disease patients can harbor an expansion of E. coli, C. tropicalis, and S. marcescens, which together have the ability to form polymicrobial biofilms *in vitro* (133). These studies thus underline that the interactions between E. coli and *Candida* species and their effect on pathogenesis are complex and strain dependent. C. albicans allows the growth of the strict anaerobe C. difficile under aerobic culture conditions (144). The ability of C. albicans to protect anaerobic bacteria under aerobic conditions is due to the rapid reduction of dissolved oxygen in the vicinity of the yeast (145). When examined in a mouse model of infection, C. albicans enhanced C. difficile pathogenicity when delivered orally 1 day prior to C. difficile infection (146). Another study found that the colonization of mice with C. albicans 3 weeks before C. difficile infection protected mice from infection (147). These two different experimental setups and outcomes indicate that the effect C. albicans has on C. difficile infection is dependent on the colonization state of C. albicans. Studies have also shown an interaction between C. albicans and H. pylori in gastric biopsy samples, where H. pylori was found within vacuoles in C. albicans cells (148, 149). It has been suggested that this behavior provides an environment that H. pylori can use to survive the low pH of the stomach (150) (Fig. 2). By analyzing whole stomachs of mice, Mason and colleagues show that antibiotic treatment allows for C. albicans colonization, triggering inflammation and inhibiting recolonization by commensal *Lactobacillus* strains (151). Alternatively, the commensal yeast S. cerevisiae enhances the growth of the opportunistic pathogen Acinetobacter baumannii by producing ethanol. Furthermore, ethanol-stimulated A. baumannii shows enhanced pathogenicity in a Caenorhabditis elegans model of infection (152). It is important to keep these potentially detrimental interactions between pathogens, opportunistic pathogens, commensal bacteria, and fungi in mind when designing therapeutics involving probiotics.

### Antagonistic interactions between pathogens.

There are also several antagonistic interactions between intestinal pathogens that do not have a clear benefit for intestinal health. One example is observed with S. marcescens, which employs a type VI secretion system to deliver antifungal toxins that kill both the yeast and hyphal form of C. albicans in liquid culture (153) (Fig. 2). *S.* Typhimurium also demonstrates a similar antifungal behavior by injecting type III secretion system effectors into C. albicans, blocking hyphal formation during C. elegans infection (154, 155). A. baumannii also demonstrates antifungal activity by binding to C. albicans filaments via OmpA and inducing apoptosis, preventing biofilm formation on polystyrene plates and limiting infection of C. elegans (156, 157). Conversely, C. albicans seems to express a mechanism to limit A. baumannii growth *in vitro* by producing the quorum-sensing molecule farnesol during late-stage biofilm formation (157). The previously mentioned symbiotic interaction provided by C. albicans to C. difficile is not reciprocated. The same study that found that C. albicans provides C. difficile with the means to grow under aerobic conditions also found that C. difficile inhibits C. albicans hyphal growth through the secretion of the small molecule *p*-cresol (144) (Fig. 2). These studies highlight bidirectional antagonistic interactions between pathogenic species of bacteria and fungi that are relevant to human health.

## MYCOBIOTA AND IMMUNE SYSTEM INTERACTION

All bacterial-fungal interactions within the host occur in an environment that is ultimately regulated by the host immune response. The immunological changes stimulated by a specific microbial colonizer can have a profound effect on the intestinal environment, affecting a wide variety of microbial species already present. This is illustrated, for example, in a study that found that Bacteroides thetaiotaomicron stimulates expression of the innate immune genes encoding hypoxia-inducible factor 1 alpha (HIF-1α) and the antimicrobial peptide LL-37-CRAMP, and this differential expression provides colonization resistance against C. albicans in mice (158). Numerous studies have been performed focusing on the impact of both bacterial and fungal species on the host immune system and vice versa, as summarized previously (104, 159–165). We will focus on how the immune system recognizes fungi and some of the most recent studies on mycobiota and immune system interactions.

### Recognition of fungi by the immune system.

The prerequisite for the host to respond to fungi is the ability of cells, in particular, immune cells, to identify and respond to different molecular patterns present on fungi. Among the pattern recognition receptors (PRRs) that can identify fungi are Toll-like receptors (TLRs), C-type lectin receptors (CLRs), and NOD-like receptors (NLRs). Fungal structures that are recognized by PRRs include surface polysaccharides, such as mannans or mannoproteins (TLR2, TLR4, Dectin-2, Mincle, and DC-SIGN), β-glucans (TLR2, Dectin-1, and NKp30), and unmethylated DNA (TLR9). Phagocytosed fungi can also activate NLRs, which leads to inflammasome formation and the production of the inflammatory protein interleukin 1 beta (IL-1β) (109, 166–169). Mutations in the receptors highlight the importance of proper recognition of fungi by the immune system. Mutations in the gene encoding Dectin-1 have been associated with increased C. tropicalis invasion in mice and exacerbated colitis in both mice and humans (12). Mutations in Dectin-2 were found to be associated with increased Candida glabrata infections due to a deficient immune response to the fungus (170). Lack of TLR4 and TLR2 responses were shown to affect disseminated candidiasis in mice: lack of TLR4 caused an increased C. albicans kidney burden, while blocking of TLR2 inhibited the production of inflammatory cytokines such as tumor necrosis factor alpha (TNF-α) and IL-1β (171). Several other receptors are involved in the recognition of fungi. These include the recently identified MelLec, a CLR able to bind melanin on A. fumigatus conidia (172), soluble receptors such as pentraxins and mannose-binding lectin (MBL), involved in the recognition of galactomannan and mannan, respectively, and the intracellular RIG-I-like receptor (RLR) MDA5, which was found to be involved in the immune response to systemic C. albicans infection (167, 173, 174).

### Immune system-mycobiota interaction.

Much emphasis has been placed on understanding the roles of the mycobiota in shaping the immune system. A recent study highlighted the important contribution of fungi in the maturation of the immune system. The authors showed that fungi colonizing the guts of mice kept in a natural outdoor environment were sufficient to induce an increase in circulating granulocytes to a level more similar to that in humans than in laboratory mice (43). This finding expanded on results showing that mice colonized with a “wild-mouse microbiota” would respond to immunotherapy in a manner more similar to that in humans (175). Fungi are not only important during homeostatic conditions but also necessary for the development of a healthy immune system. Most of the research performed tries to understand the involvement of the mycobiota in the development and origin of inflammatory and pathogenic conditions. *Candida* and *Malassezia* are among the fungal genera that have been most studied in this context. *Candida* species, particularly C. albicans, are known to be able to exacerbate gut inflammation (161). Fungal dysbiosis and increased C. albicans colonization were identified in association with IBD in human patients (176). In a mouse model of DSS-induced colitis, the presence of C. albicans also worsened local and systemic inflammation (177). Like C. albicans, *M. restricta* was shown to increase disease severity in DSS-treated mice. Increased relative abundance of *M. restricta* in the colons of Crohn’s disease patients was linked to a mutation in the CARD9 gene, CARD^S12N^, previously associated with the onset of IBD (105). This ability of *Malassezia* to elicit inflammation was later connected to the activation of the NLRP3 inflammasome (178). Dysbiosis of the gut mycobiota can also affect distal organs. A recent study found that gut dysbiosis, specifically, increased abundance of *Malassezia* species, promoted pancreatic ductal adenocarcinoma development through the activation of the complement cascade via the engagement of the mannose-binding lectin (33). Gut dysbiosis has also been linked to lung inflammation. During gut inflammation, C. albicans was shown to induce the generation of Th17 cells cross-reactive against the airborne pathogen A. fumigatus, thus contributing to the exacerbation of allergic bronchopulmonary aspergillosis (179). Similarly, CX3CR1^+^ mononuclear phagocytes, present in the lamina propria and involved in trafficking bacteria from the gut to mesenteric lymph nodes (180), were identified to play an important role in the immunity against fungi in the gut (181) and are able to create a gut-lung axis that exacerbates allergic airway disease following gut fungal dysbiosis (182). Another example of interconnection between the gut and lungs is the expansion of Wallemia mellicola in the gut following antibiotic treatment. This intestinal expansion exacerbates lung inflammation by increasing eosinophil recruitment in a mouse model of allergic airway disease (44). A recently published study finally highlighted the importance of a balanced immune response to control fungal infections. Th17 immune responses are known to be important during mucocutaneous fungal infections, especially C. albicans infections (162). However, Break and colleagues showed that in mice and human patients with mutation in the gene *Aire* that had an intact Th17 response, excessive gamma interferon (IFN-γ) production by T cells at the mucosal level was the cause of increased susceptibility to chronic mucocutaneous candidiasis (183). Future studies will continue to expand our knowledge on the role of fungi during homeostatic and pathological conditions and dissect their role in the development of a balanced immune system.

## CONCLUSION AND OUTLOOK

Mycobiome research is a rapidly expanding scientific field, but many questions are currently still unanswered. Due to the high inter- and intraindividual variability, it is unclear if core mycobiomes can be defined. Future research will expand our knowledge on which fungi are resident and which are transiently present in the gastrointestinal tract. However, it is indisputable that the mycobiome fulfills crucial roles. Irrespective of their ability to colonize, fungi interact with and train the immune system and contribute to gastrointestinal homeostasis. Analogous to bacteriome research, mycobiome research is also moving from describing composition to ascribing function. Highly interesting mechanisms of how specific gut commensal fungi modulate immune responses and interact with bacteria are beginning to emerge. Future research directions include the characterization of the fungal metabolome in the gastrointestinal tract to identify which products are produced by fungi and how they influence the microbiome and the host. A recent analysis of the metabolome of differently colonized gnotobiotic mice found that fungi significantly contributed to microbial ecology and host immune functionality but contributed only a small extent to the overall gut metabolome (175). More research with an extended spectrum of commensal gut fungi and additional models will be needed to define the fungal metabolome and the role of fungi in the gut ecosystem.
